# Comparison of intervention effects in split-mouth and parallel-arm randomized controlled trials: a meta-epidemiological study

**DOI:** 10.1186/1471-2288-14-64

**Published:** 2014-05-11

**Authors:** Violaine Smaïl-Faugeron, Hélène Fron-Chabouis, Frédéric Courson, Pierre Durieux

**Affiliations:** 1 Institut National de la Santé et de la Recherche Médicale, U1138, Equipe 22, Centre de Recherche des Cordeliers, Paris, France; 2 Assistance Publique-Hôpitaux de Paris, Hôpital Bretonneau, Service d’Odontologie, Paris, France; 3 Université Paris Descartes - Sorbonne Paris Cité, Faculté de Chirurgie Dentaire, Unité de Recherches Biomatériaux Innovants et Interface EA4462 Montrouge, France; 4 Assistance Publique-Hôpitaux de Paris, Hôpital Charles Foix, Service d’Odontologie, Ivry- sur-Seine, France; 5 Université Paris Descartes - Sorbonne Paris Cité, Faculté de Médecine, Paris, France; 6 Département d’Informatique Hospitalière, Assistance Publique-Hôpitaux de Paris, Hôpital Européen Georges Pompidou, Paris, France; 7 Unité de Recherches Biomatériaux Innovants et Interface EA4462, 1 rue Maurice Arnoux, Montrouge 92120, France

**Keywords:** Meta-analysis, Randomized controlled trial, Split-mouth trial, Bias, Meta-epidemiological study

## Abstract

**Background:**

Split-mouth randomized controlled trials (RCTs) are popular in oral health research. Meta-analyses frequently include trials of both split-mouth and parallel-arm designs to derive combined intervention effects. However, carry-over effects may induce bias in split- mouth RCTs. We aimed to assess whether intervention effect estimates differ between split- mouth and parallel-arm RCTs investigating the same questions.

**Methods:**

We performed a meta-epidemiological study. We systematically reviewed meta- analyses including both split-mouth and parallel-arm RCTs with binary or continuous outcomes published up to February 2013. Two independent authors selected studies and extracted data. We used a two-step approach to quantify the differences between split-mouth and parallel-arm RCTs: for each meta-analysis. First, we derived ratios of odds ratios (ROR) for dichotomous data and differences in standardized mean differences (∆SMD) for continuous data; second, we pooled RORs or ∆SMDs across meta-analyses by random-effects meta-analysis models.

**Results:**

We selected 18 systematic reviews, for 15 meta-analyses with binary outcomes (28 split-mouth and 28 parallel-arm RCTs) and 19 meta-analyses with continuous outcomes (28 split-mouth and 28 parallel-arm RCTs). Effect estimates did not differ between split-mouth and parallel-arm RCTs (mean ROR, 0.96, 95% confidence interval 0.52–1.80; mean ∆SMD, 0.08, -0.14–0.30).

**Conclusions:**

Our study did not provide sufficient evidence for a difference in intervention effect estimates derived from split-mouth and parallel-arm RCTs. Authors should consider including split-mouth RCTs in their meta-analyses with suitable and appropriate analysis.

## Background

In split-mouth randomized controlled trials (RCTs) in oral health, experimental and control interventions are randomly allocated to different areas in the oral cavity (teeth, surfaces, arches, quadrants) [1-3]. As compared with parallel-arm RCTs, split-mouth RCTs have the advantage that most of the variability of outcome among patients is removed from the intervention effect estimate for a potential increase in statistical power, each subject being its own control [4,5]. Because every subject receives each intervention, the design may also be better suited to determine patient preferences.

Many researchers in oral health research use the split-mouth design. Therefore, systematic review authors frequently include trials of both split-mouth and parallel-group designs to derive combined intervention effects. The rationale to include split-mouth RCTs is to use all the available evidence. However, the split-mouth design may lead to biased intervention effect estimates. For instance, carry-over effects (ie, contamination or “spilling” of the effects of one intervention from one site to another site) may induce bias in split-mouth RCTs [4]. If the interventions are delivered at different times, period effects may also influence intervention effects. Moreover, the statistical analysis of split-mouth differs from that of parallel-arm RCTs because the paired nature of data must be taken into account [6-8]. Failure to consider the difference between the two types of trials may result in unreliable inference because the confidence interval for the true combined effect will be incorrect. Lesaffre et al. suggested that intervention effect estimates from split-mouth and parallel-arm RCTs may not be the same and recommended separate subgroup meta-analyses of split-mouth and parallel-arm RCTs to investigate systematic differences [9].

In this meta-epidemiological study, we aimed to assess if data from split-mouth RCTs were incorporated appropriately in meta-analyses and whether intervention effect estimates differ between split-mouth and parallel-arm RCTs in meta-analyses.

## Methods

We performed a meta-epidemiological study to compare intervention effect estimates between split-mouth RCTs and parallel-arm RCTs. We identified meta-analyses that included at least one split-mouth RCT and at least one parallel-arm RCT assessing a variety of conditions and interventions on binary or continuous outcomes. For each selected meta- analysis, we compared intervention effect estimates between split-mouth and parallel-arm RCTs. In a second stage, results were summarized across all meta-analyses.

### Selection of meta-analyses, trials and outcomes

To identify eligible studies, we searched MEDLINE and EMBASE. Search equations for each database included the free-text word “split mouth” combined with a filter designed to identify systematic reviews (see Additional file 1) [10]. Second, we performed a full-text search of the Cochrane Database of Systematic Reviews (CDSR) through http://www.thecochranelibrary.com and archie.cochrane.org. We also searched the Database of Abstracts of Reviews (DARE). Third, we searched SCIRUS, a science-specific search engine covering full-text articles. The last search was conducted in February 2013, with no restriction on date or language.

Two authors independently and in duplicate screened the titles and abstracts of records retrieved by the search, and then screened the selected full-text reports. When the designs of selected trials were unclear in an abstract, we always screened the full-text article. Any disagreements were resolved by discussion.

Eligible studies were systematic reviews of therapeutic or preventive interventions that included at least one split-mouth RCT, as labeled by the review authors, and at least one parallel-arm RCT in quantitative syntheses (ie, meta-analyses). Updates of systematic reviews were selected rather than initial versions.

From each eligible systematic review, we selected all independent meta-analyses (defined as the comparison between specific experimental and control interventions on a given outcome). We excluded meta-analyses in which all RCTs had a split-mouth or parallel-arm design. Then we selected one binary or one continuous outcome, or both if present, corresponding to the previous criteria. In cases of multiple eligible outcomes, we chose the primary outcome as stated by the authors or selected the outcome with the largest number of studies. For each meta-analysis, we selected all individual RCTs and we excluded non-randomized studies. Finally, we identified overlapping meta-analyses (ie, with common RCTs) and excluded the meta- analysis that included fewer trials [11].

### Data extraction

Two authors extracted the data in duplicate and independently, with discrepancies resolved by discussion. For each systematic review, we recorded the first author, publication year and studied population. For each meta-analysis, we recorded the experimental intervention, the comparator, the outcome and the number of split-mouth and parallel-arm RCTs.

We assessed the methods used by the authors for incorporating split-mouth RCTs into meta- analyses: we assessed the presence of subgroup analyses (ie, split-mouth RCTs and parallel-arm RCTs analyzed separately) and/or whether one quantitative synthesis combined split-mouth and parallel-arm RCTs; in this case, we assessed whether the techniques described by Elbourne 2002 or Lesaffre 2009 were used [9,12]; moreover, we assessed whether the authors calculated the standard error of the intervention effect estimate in split- mouth RCTs using appropriate methods (ie, statistical approaches taking into account the paired nature of data; eg, the techniques described by Follmann) [13].

From the systematic review, for each RCT, we abstracted the first author and publication year and the design (split-mouth or parallel-arm). From the original RCT reports, we extracted the number of patients and, according to type of outcome, the means and associated SDs, or number of events, for both the experimental and control arms.

### Statistical analysis

Binary and continuous outcomes were analyzed separately. For each RCT, we derived an intervention effect estimate and the associated sampling variance. Intervention effects were measured by odds ratios (ORs) and standardized mean differences (SMDs, or Cohen’s *d*). All comparisons were coded so that the experimental intervention was compared with the comparator for an unfavorable outcome. Binary and continuous outcomes were coded so that an OR < 1 and SMD < 0 indicated a beneficial effect of the experimental intervention, respectively. For binary outcomes, in cases of 0-count cells, we used a 0.5 continuity correction. If no events occurred in all arms, the RCT was excluded.

For split-mouth RCTs, we took into account the matched nature of data; marginal ORs were calculated by the method of Becker and Balagtas [12,14,15]; SMDs were estimated by taking into account the within-patient correlation coefficient [16]. We contacted the corresponding authors of all split-mouth RCTs to ask for the required matched outcome data. If not available from the reports and with no response from authors, we assumed a within- patient correlation of 0.5. Sensitivity analyses with correlation values of 0 and 0.25 yielded similar results.

For every meta-analysis, with more than one split-mouth or parallel-arm RCT, we calculated combined intervention effects and associated variances. We used both fixed-effects and random-effects (restricted maximum likelihood estimator) calculations. Results were similar, so we reported results obtained with fixed-effects primarily and those obtained with random-effects as sensitivity analysis.

We used a meta-epidemiological analysis to estimate the combined difference in intervention effect estimates between split-mouth and parallel-arm RCTs by a two-step method [17]. For each meta-analysis with a binary outcome, we estimated the ratio of the intervention effect for split-mouth RCTs to that for parallel-arm RCTs, the ratio of ORs (ROR): on a logarithmic scale, we derived log(ROR) = log(summary OR in split-mouth RCTs) - log(summary OR in parallel-arm RCT) and its variance Var(log ROR) = Var[log(summary OR in split-mouth RCTs)] + Var[(log summary OR in parallel-arm RCTs)]. Then we estimated a combined ROR and 95% confidence interval (CI) across meta-analyses by a random-effects meta-analysis model (restricted maximum likelihood estimator). For each meta-analysis with a continuous outcome, we estimated the difference in intervention effect estimates between split-mouth and parallel-arm RCTs, the difference in SMDs (∆SMD): we derived ∆SMD = summary SMD in split-mouth RCTs - summary SMD in parallel-arm RCT and its variance Var(∆SMD) = Var[summary SMD in split-mouth RCTs] + Var[summary SMD in parallel-arm RCTs]. Then we estimated a combined ∆SMD across meta- analyses and 95% CI across meta-analysis by a random-effects meta-analysis model (restricted maximum likelihood estimator). An ROR < 1 or ∆SMD < 0 indicated that split-mouth RCTs yielded larger intervention effect estimates than parallel-arm RCTs. Heterogeneity in RORs or ∆SMDs across the different meta-analyses was assessed by the I^2^ statistic and tau^2^ the between-meta-analyses variance. We plotted the results on forest plots. We reported the 95% prediction intervals for the ROR and ∆SMD, respectively, which provide a predicted range for the true difference in treatment effects between split-mouth and parallel-arm RCTs in an individual meta-analysis.

Analyses involved use of the R software (online at http://www.R-project.org, the R Foundation for Statistical Computing, Vienna,Austria). A 2-tailed P < 0.05 was considered statistically significant.

## Results

### Eligible systematic reviews and meta-analyses

**Figure 1:**
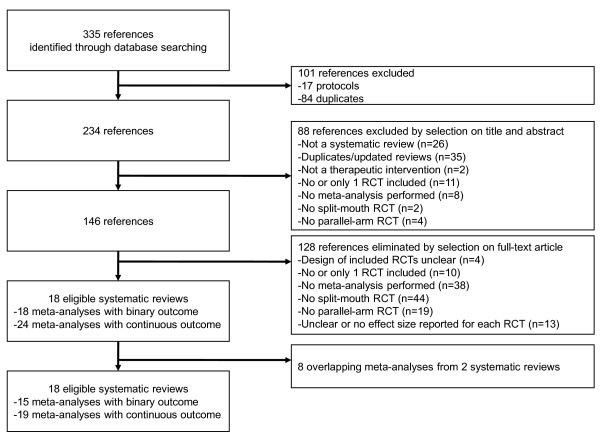
Flow diagram.

The search yielded 335 potentially eligible articles. The flow chart of selection and reasons for exclusion are in Figure 1. We included 18 systematic reviews [18-36]; 8 were Cochrane systematic reviews [23,25-27,30,33,34,36]. The selected reviews were all published recently (range 2006 to 2013). The reviews concerned interventions for periodontal disease (n = 9), dental surgery/implantology (n = 6), dental caries (n = 2), and orthodontic treatment (n = 1) (Table 1).

**Table 1:** 18 selected systematic reviews

**Review**	**Population**
1. Annibali [18]	Patients treated with dental implants
2. Atieh [19]	Patients with a clinical diagnosis of chronic periodontitis
3. Brignardello-Petersen [20]	Patients who underwent surgical removal of impacted mandibular third molars
4. Cairo [21]	Patients with a clinical diagnosis of Miller Class I or II localized gingival recession defect
5. Carrasco-Labra [22]	Healthy adult subjects who underwent surgical removal of an impacted mandibular third molar
6. Chambronne [23]	Patients with gingival recession areas (Miller’s Class I or II > 3 mm)
7. Del Fabbro [24]	Patients undergoing surgical procedures for the treatment of periodontal defects and gingival recession
8. Esposito [25]	Patients with osteointegrated root-form dental implants
9. Esposito [26]	Patients with chronic, aggressive, or early onset periodontitis and intrabony defects with an intrabony component of at least 3 mm to be treated
10. Esposito [27]	Patients rehabilitated with implant supported/retained prostheses
11. Fleming [28]	Patients with full-arch, fixed, and bonded orthodontic appliances
12. Imai [29]	Adult patients with clinical signs of gingivitis and some periodontitis
13. Lodi [30]	Patients undergoing tooth extraction for any indication
14. Mickenautsch [31]	Patients requiring dental restoration
15. Muller-Bolla [32]	Patients > 5 years old with permanent molars, all caries-free or with incipient carious lesions
16. Needleman [33]	Patients ≥ 21 years old with chronic periodontitis or periodontitis
17. Sgolastra [35]	Adult patients with chronic periodontitis
18. Yong [36]	Adult patients with adrenal insufficiency and who required surgery

From the 18 systematic reviews, 42 meta-analyses were eligible. The identification of overlapping meta-analyses led to the exclusion of 8 meta-analyses (from 2 systematic reviews). Consequently, 34 meta-analyses contributed to our analysis: 15 with binary outcome data, and 19 with continuous outcome data. The median number of RCTs per meta- analysis was 4 (range 2–16) (Table 2).

**Table 2:** 35 selected meta-analyses (15 with binary outcomes and 19 with continuous outcomes)

**Meta-analysis**	**Experimental intervention**	**Comparator**	**Binary outcome**	**Continuous outcome**
1	Platform-switched implant restoration	Platform match	-	Marginal bone loss
2	Scaling and root planning + antimicrobial photodynamic therapy	Scaling and root planning	-	Clinical attachment level gain
3.	Low-level laser energy irradiation	Nonactive comparator	-	Trismus
4.a.	Coronally advanced flap + connective tissue graft	Coronally advanced flap	Complete root coverage	Gingival recession change
4.b.	Coronally advanced flap + enamel matrix derivative	Coronally advanced flap	Complete root coverage	Gingival recession change
4.c.	Coronally advanced flap + acellular dermal matrix	Coronally advanced flap	Complete root coverage	Gingival recession change
4.d.	Coronally advanced flap + barrier membranes	Coronally advanced flap + connective tissue graft	Complete root coverage	Gingival recession change
4.e	Coronally advanced flap + acellular dermal matrix	Coronally advanced flap + connective tissue graft	Complete root coverage	Gingival recession change
5.	Secondary closure technique	Primary closure technique	Infectious complication	Pain
6.a	Guided tissue regeneration (rm) + bone substitutes	Subepithelial connective tissue grafts	-	Gingival recession change
6.b	Guided tissue regeneration (rm) + bone substitutes	Guided tissue regeneration (rm)	Complete root coverage	Gingival recession change
7.	Platelet-rich plasma	Control	-	Clinical attachment level gain
8.a	Loading of osteointegreated implants within 1 week	Loading of osteointegreated implants after 2 months	Prosthesis failure	Marginal bone level changes
8.b	Loading of osteointegreated implants within 1 week	Loading of osteointegreated implants between 1 week and 2 months	Prosthesis failure	Marginal bone level changes
9.	Emdogain	Control	Probing attachment level gain < 2 mm	-
10.	Procedure with flap elevation	Flapless implant insertion	Implant failure	-
11.	1-stage bonding (self-etch)	2-stage bonding (acid-etch)	Bracket failure	-
12.	Dental floss	Interdental brushes	-	Interproximal gingival bleeding
13.	Antibiotic - Pre-operative prophylaxis	Placebo	-	Pain
14.	Glass-ionomer cement	Amalgam	Caries	-
15.a	Fluoride-containing resin-based sealant	Light-cured resin-based sealant	Complete retention	-
15.b	Rubber dam	Cotton rolls	Complete retention	-
16	Guided tissue regeneration	Control	-	Clinical attachment level gain
17.	Scaling and root planning + Diode laser	Scaling and root planning	-	Probing depth reduction
18.	Supplemental perioperative steroids	Placebo	-	Systolic blood pressure

rm: with resorbable membranes.

### Methods used for incorporating split-mouth trials into meta-analyses

In all systematic reviews, the authors combined split-mouth trials together with parallel-arm trials in meta-analyses (Table 3). For 6 of 18 systematic reviews, the authors also meta- analyzed split-mouth and parallel-arm trials separately in subgroup analyses. Regarding the standard error of the intervention effect estimate in split-mouth RCTs, in 8 reviews, how the paired nature of data was taken into account was not clear and in another 8 reviews, the paired nature of data was imputed with methods described by Follmann [13] when the appropriate data were not present in RCT reports. Finally, we contacted the authors of all split-mouth RCTs to ask for the matched outcome data and we received 16 responses.

**Table 3:** Methods used by review authors to incorporate split-mouth RCTs into meta-analyses

**Review**	**Split-mouth and parallel arm RCTs combined**		**Standard error of the treatment effect estimate in split-mouth RCTs**
	**Together**	**Separately (subgroups)**	
1. Annibali [18]	Yes	Yes	Imputed using Follmann [13], with the appropriate data not presented
2. Atieh [19]	Yes	No	Not clear
3. Brignardello-Petersen [20]	Yes	Yes	Within-patient correlation assumed equal to 0
4. Cairo [21]	Yes	No	Imputed using Follmann [13], with the appropriate data not presented
5. Carrasco-Labra [22]	Yes	Yes	Imputed using Follmann [13], with the appropriate data not presented. Within-patient correlation assumed equal to 0.75
6. Chambronne [23]	Yes	Yes	Imputed using Follmann [13], with the appropriate data not presented
7. Del Fabbro [24]	Yes	No	Not clear
8. Esposito [25]	Yes	No	Imputed using Follmann [13], with the appropriate data not presented
9. Esposito [26]	Yes	Yes	Imputed using Follmann [13], with the appropriate data not presented. Within-patient correlation assumed equal to 0.25 (median ICC in similar review, Needleman [33])
10. Esposito [27]	Yes	No	Imputed using Follmann [13], with the appropriate data not presented
11. Fleming [28]	Yes	No	Calculated using Borenstein [16]
12. Imai [29]	Yes	No	Not clear
13. Lodi [30]	Yes	No	Not clear
14. Mickenautsch [31]	Yes	No	Not clear
15. Muller-Bolla [32]	Yes	No	Not clear
16. Needleman [33]	Yes	Yes	Imputed using Follmann [13], with the appropriate data not presented. Within-patient correlation assumed equal to 0.25
17. Sgolastra [35]	Yes	No	Not clear
18. Yong [36]	Yes	No	Not clear

### Characteristics of split-mouth and parallel-arm trials

The15 meta-analyses with binary outcome data involved 28 split-mouth and 28 parallel-arm RCTs; the19 meta-analyses with continuous outcome data involved 45 split-mouth and 48 parallel-arm RCTs, for 56 and 65 distinct split-mouth and parallel-arm RCTs, respectively. Parallel-arm RCTs were published later than split-mouth RCTs (median [25%-75% percentile] 2007 [2002–2008] versus 2004 [1999–2008]); the first published RCT was a split-mouth RCT in 20 of the 34 meta-analyses. Parallel-arm RCTs had a larger median sample size than split-mouth RCTs (median 40 [29–90] versus 20 [12-30]).The median total relative weight of split-mouth RCTs in each meta-analysis was 51% [39–71%] for the 34 meta-analyses.

### Differences in intervention effect between split-mouth and parallel-arm trials

**Figure 2:**
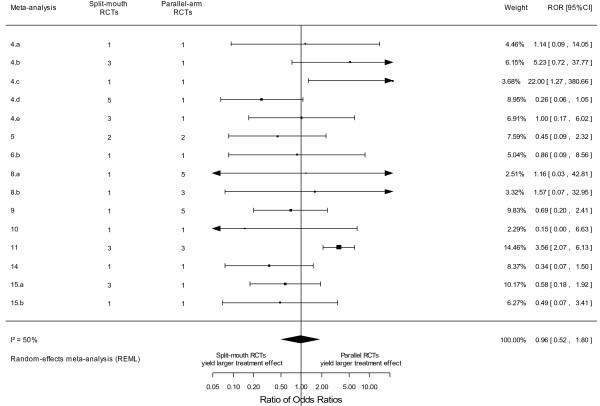
Difference in intervention effect estimates between split-mouth and parallel-arm randomized controlled trials for binary outcome data.

Among the 15 meta-analyses with binary outcome data, 8 yielded a larger intervention effect for split-mouth RCTs (none with evidence for a difference between the two estimates) and 6 a larger intervention effect for parallel-arm RCTs (2 with evidence for a difference between the two estimates) (see Additional file 2). Split-mouth and parallel-arm RCTs did not differ in intervention effect estimates: the meta-epidemiological analysis yielded a combined ROR of 0.96 (95% CI 0.52–1.80, p = 0.91, I^2^ = 50%, 95% CI 9%–80%, and tau^2^ = 0.62 across meta-analyses) (Figure 2). The associated 95% prediction interval for the ROR was 0.19 to 5.08. Finally, when using random-effects models for within-meta-analysis calculations of summary effect sizes in split-mouth and parallel-arm RCTs, it yielded a combined ROR of 0.79 (95% CI 0.47–1.32, p = 0.36).

**Figure 3:**
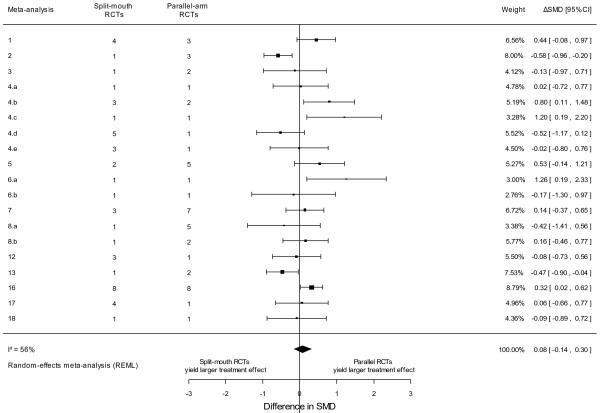
Difference in intervention effect estimates between split-mouth and parallel-arm randomized controlled trials for continuous outcome data.

Among the 19 meta-analyses with continuous outcome data, 8 yielded a larger intervention effect for split-mouth RCTs (2 with evidence for a difference between the two estimates) and 9 a larger intervention effect for parallel-arm RCTs (4 with evidence for a difference between the two estimates) (see Additional file 2). Split-mouth and parallel- arm RCTs did not differ in intervention effect estimates: the meta-epidemiological analysis yielded a combined ∆SMD of 0.08 (95% CI -0.14–0.30, p = 0.46, I^2^ = 56%, 95% CI 21%–82%, and tau^2^ = 0.12 across meta- analyses) (Figure 3). The associated 95% prediction interval for the ∆SMD was -0.63 to 0.79. Finally, when using random-effects models for within-meta-analysis calculations of summary effect sizes in split-mouth and parallel-arm RCTs, it yielded a combined ∆SMD of 0.05 (95% CI -0.21–0.30, p = 0.73).

In all, 6 of 8 meta-analyses showing differences between split-mouth and parallel-arm RCTs beyond chance did not meta-analyze split-mouth and parallel-arm RCTs separately in subgroup analyses.

## Discussion

In our meta-epidemiological study, we found that split-mouth trials contributed half of the evidence in meta-analyses. Contrary to the recommendations by Lesaffre et al. and the Cochrane Oral Health group, most systematic reviews did not meta-analyze split-mouth and parallel-arm trials separately in subgroup analyses [37]. Moreover, most reviews did not report explicitly how split-mouth RCTs were handled in meta-analyses, while others approximated a paired analysis by imputing within-patient correlations. Finally, our meta- epidemiological study did not provide sufficient evidence for a systematic difference in intervention effect estimates between split-mouth and parallel-arm RCTs, both for continuous and binary outcome data.

The main difference between split-mouth and parallel-arm trials with regard to mechanisms of bias is that, in split-mouth trials, interventions may have effects on parts of the dentition other than those to which they were assigned; these carry-over effects put split-mouth trials at risk of bias. However, no method exists to assess or test the extent of carry-across effects in a split-mouth trial. As a consequence, the possibility of carry-over effects should be considered before deciding on whether a split-mouth design should be used. As far as we can judge a posteriori, the effects of interventions assessed in the reviews selected for our meta-epidemiological study were always localized.

Previous meta-epidemiological studies showed that individual study processes (eg, inadequate allocation concealment, non-blinding [38]) or nonprocess-related factors (eg, whether a study was conducted at a single center [39]) may put a randomized trial at risk of bias [40]. Very few meta-epidemiological studies have assessed if study designs itself could be associated with treatment effect estimates. Lathyris et al. focused on the cross-over design, which is relevant to oral health research and biomedical research in general; the results of crossover trials tended to agree with those of parallel-arm trials [41]. Here, we focused on the split-mouth design, which is popular in oral health research. This type of design is in fact also relevant to other fields of biomedical research in general, in which split- body studies allocate the interventions to separate parts of the body of each participant. However, these trials are infrequent (about 2-3% of randomized trials indexed in Pubmed) [42,43] and we could find only one meta-analysis including at least one split-body trial and at least one parallel-arm trial [44].

Our findings are based on recently published systematic reviews covering a fair range of conditions and interventions in oral healthcare. Consequently, our results are more generalizable than could be obtained with focus on a particular topic. Our study has several limitations. We selected a relatively small number of systematic reviews for our meta-epidemiological study. It is difficult to identify reviews with both parallel-arm and split-mouth trials with usual strategies and we acknowledge that unidentified reviews may exist. However, we systematically searched for both Cochrane and non-Cochrane reviews, including a search of full-text articles indexed in the Cochrane library and in the Scirus database. Unfortunately, the latter service is no longer running. We acknowledge that searching additional regional databases (e.g., LILACS, PASCAL) and full-text databases (e.g., HighWire Press, Google Scholar) may be very useful to identify potentially eligible systematic reviews. Eligible reviews may be missing because of reporting bias (including location bias and language bias). However, reporting bias is usually driven by the magnitude/direction and statistical significance of treatment effects. We see no reason for reviews to be missing because of the difference in treatment effect estimates between split-mouth and parallel-arm RCTs. As a consequence, the impact of missing reviews is unpredictable and probably limited on our meta-epidemiological study. On top of the relatively small number of selected reviews, the number of split-mouth and parallel-arm RCTs in each meta-analysis was small. Meta-analyses typically include a limited number of trials: the median number of trials in a large sample of Cochrane meta- analyses was 3 [45]. The consequence is uncertainty in the difference between the 2 study designs. Because of these limitations, and as it is to our knowledge the first meta- epidemiological investigation on the subject, we acknowledge that these results should be replicated, by including additional comparisons between the two designs as they become available. A second caveat is that we did not assess risk of bias within each RCT and we cannot assess meta-confounding. The split-mouth and parallel-arm trials in the selected reviews may differ in their methodological quality. However, it would be difficult to assess the risk of bias in selected split-mouth trials because assessing internal validity requires adequate reporting and split-mouth trials frequently exhibit poor or inadequate reporting [37]. Moreover, meta-confounding because of systematic differences in risk of bias between split- mouth and parallel-arm trials would be an alternative explanation for an association between trial design and treatment effect estimates but we did not find evidence of such an association.

Our results support the use of all available evidence in systematic reviews, including that from split-mouth and parallel-arm RCTs, and authors should consider results from both trial designs in syntheses of oral health primary research. The incorporation of split-mouth RCTs should follow adequate methods [9,12]; moreover, for each split-mouth RCT, the difference between groups rather than estimates per group must be used and the standard error of the intervention effect estimate should take the matched nature of data into account [13].

Because trials in this field are frequently small, one should not be confident that the true intervention effect lies closer to the effect estimates from parallel-arm or split-mouth trials. Even when combining split-mouth and parallel-arm RCTs in the same meta-analysis, consideration should be given to potential differences between the different types of trials in subgroup analyses, until there is more evidence that the two designs do not systematically differ. Meta- analysts should also consider issues of external validity because split-mouth trials include patients with symmetrical caries or lesions who could differ from other patients in terms of possibly poorer brushing and dietary behavior.

## Conclusions

Our meta-epidemiological study did not provide sufficient evidence for a difference in intervention effect estimates derived from split-mouth and parallel-arm RCTs. Systematic review authors should consider including split-mouth RCTs in their meta-analyses with suitable and appropriate analysis.

## Abbreviations

RCT: Randomized controlled trial OR, Odds ratio; SMD: Standardized mean difference ROR, Ratio of odds ratios; ∆SMD: Difference in standardized mean differences CI, Confidence interval.

## Competing interests

The authors declare that they have no competing interests.

## Authors’ contributions

VSF provided substantial contributions to conception and design of the study, data extraction, analysis and interpretation of data, drafted the article and revised it critically for important intellectual content. HFC provided substantial contributions to acquisition of data and interpretation of data, revised the article critically for important intellectual content. FC provided substantial contributions to interpretation of data and revised the article critically for important intellectual content. PD provided substantial contributions to conception and design of the study, interpretation of data and revised the article critically for important intellectual content. All authors read and approved the final manuscript.

## Pre-publication history

The pre-publication history for this paper can be accessed here:

http://www.biomedcentral.com/1471-2288/14/64/prepub

## Supplementary Material

Additional file 1 Electronic search strategy. Click here for file

Additional file 2 Comparisons of the summary ORs and SMDs between split-mouth and parallel-arm RCTs in each meta-analysis. Click here for file
