# Erratum to: “Comparison of intervention effects in split-mouth and parallel-arm randomized controlled trials: a meta-epidemiological study”

**DOI:** 10.1186/s12874-015-0056-4

**Published:** 2015-09-02

**Authors:** Violaine Smaïl-Faugeron, Hélène Fron-Chabouis, Frédéric Courson, Pierre Durieux

**Affiliations:** Institut National de la Santé et de la Recherche Médicale, U1138, Equipe 22, Centre de Recherche des Cordeliers, Paris, France; Assistance Publique-Hôpitaux de Paris, Hôpital Bretonneau, Service d’Odontologie, Paris, France; Université Paris Descartes - Sorbonne Paris Cité, Faculté de Chirurgie Dentaire, Unité de Recherches Biomatériaux Innovants et Interface, EA4462 Montrouge, France; Assistance Publique-Hôpitaux de Paris, Hôpital Charles Foix, Service d’Odontologie,, Ivry- sur-Seine, France; Université Paris Descartes - Sorbonne Paris Cité, Faculté de Médecine, Paris, France; Département d’Informatique Hospitalière, Assistance Publique-Hôpitaux de Paris, Hôpital Européen Georges Pompidou, Paris, France; Unité de Recherches Biomatériaux Innovants et Interface EA4462, 1 rue Maurice Arnoux, 92120 Montrouge, France

## Erratum

The original version of this article unfortunately contained a mistake in the Abstract. The correct information is given below.

“We selected 18 systematic reviews, for 15 meta-analyses with binary outcomes (28 split-mouth and 28 parallel-arm RCTs) and 19 meta-analyses with continuous outcomes (45 split-mouth and 48 parallel-arm RCTs).”

## References

[1] Smaïl-Faugeron S, et al. Comparison of intervention effects in split-mouth and parallel-arm randomized controlled trials: a meta-epidemiological study. BMC Medical Research Methodology. 2014;14:64. 10.1186/1471-2288-14-64 PMC4023173 24886043

