# Supplementary material for: Comparison of intervention effects in split-mouth and parallel-arm randomized controlled trials: a meta-epidemiological study
Source: BMC Med Res Methodol. 2014 May 11;14:64. doi: 10.1186/1471-2288-14-64 (PMC4023173; doi:10.1186/1471-2288-14-64)
Supplement: Additional file 1 — Electronic search strategy. [file 1471-2288-14-64-S1.doc]

**Comparison of intervention effects in split-mouth and parallel-arm randomized controlled trials: a meta-epidemiological study**

Supplementary appendix A1. Electronic search strategy

Date of search: February 11^th^, 2013

MEDLINE via Pubmed

"split mouth" AND (("systematic review" OR meta-analysis OR meta-analyses) OR ("Cochrane database of systematic reviews (Online)"[Jour]) OR (systematic[sb]) OR ("split mouth" AND (MEDLINE OR "systematic review" OR meta-analysis[Publication Type] OR intervention*[ti])))

EMBASE

('split mouth' AND ('medline'/exp OR 'systematic review'/exp OR 'meta-analysis'/exp OR intervention*:ti) AND [embase]/lim) OR ('split mouth' AND ([meta analysis]/lim OR [systematic review]/lim) AND [embase]/lim) OR ('split mouth' AND ('systematic review'/exp OR 'meta analysis'/exp OR 'meta analyses') AND [embase]/lim)

ARCHIE Cochrane

Text contains split-mouth - Document type Review Document - Review stage Full review - Review status Active - Review Type Intervention review

Cochrane Database of Systematic Reviews

Search all text “split mouth”

SCIRUS

(title:"systematic review" OR title:"meta-analysis") ("split-mouth")
