# Supplementary material for: Comparison of intervention effects in split-mouth and parallel-arm randomized controlled trials: a meta-epidemiological study
Source: BMC Med Res Methodol. 2014 May 11;14:64. doi: 10.1186/1471-2288-14-64 (PMC4023173; doi:10.1186/1471-2288-14-64)
Supplement: Additional file 2 — Comparisons of the summary ORs and SMDs between split-mouth and parallel-arm RCTs in each meta-analysis. [file 1471-2288-14-64-S2.doc]

# **Comparison of intervention effects in split-mouth and parallel-arm randomized controlled trials: a meta-epidemiological study**

Supplementary appendix A2. Comparisons of the summary ORs and SMDs between split-mouth and parallel-arm RCTs in each meta-analysis.

Summary effect sizes were estimated with fixed-effects meta-analysis. Data in blue indicate meta-analyses for which the difference between summary ORs/SMDs in split-mouth RCTs and in parallel-arm RCTs was beyond what would be expected by chance alone. OR<1 or SMD<0 favors experimental treatment and OR >1 or SMD>0 favors the control treatment.
